# The Lognormal Lung: A new approach to quantifying lung inhomogeneity in COPD

**DOI:** 10.3389/fphys.2022.1032126

**Published:** 2022-10-31

**Authors:** Nicholas M. J. Smith, Snapper R. M. Magor-Elliott, Christopher J. Fullerton, John H. Couper, Graham Richmond, Gus Hancock, Grant A. D. Ritchie, Peter A. Robbins, Nick P. Talbot, Nayia Petousi

**Affiliations:** ^1^ Department of Chemistry, Physical and Theoretical Chemistry Laboratory, University of Oxford, Oxford, United Kingdom; ^2^ Department of Physiology, Anatomy and Genetics, University of Oxford, Oxford, United Kingdom; ^3^ Oxford NIHR Biomedical Research Centre, University of Oxford, Oxford, United Kingdom; ^4^ Nuffield Department of Medicine, University of Oxford, Oxford, United Kingdom

**Keywords:** COPD-chronic obstructive pulmonary disease, respiratory physiology, lung inhomogeneity, respiratory disease, translational physiology

## Abstract

Early diagnosis and disease phenotyping in COPD are currently limited by the use of spirometry, which may remain normal despite significant small-airways disease and which may not fully capture a patient’s underlying pathophysiology. In this study we explored the use of a new non-invasive technique that assesses gas-exchange inhomogeneity in patients with COPD of varying disease severity (according to GOLD Stage), compared with age-matched healthy controls. The technique, which combines highly accurate measurement of respiratory gas exchange using a bespoke molecular flow sensor and a mechanistic mathematical model of the lung, provides new indices of lung function: the parameters σCL, σCd, and σVD represent the standard deviations of distributions for alveolar compliance, anatomical deadspace and vascular conductance relative to lung volume, respectively. It also provides parameter estimates for total anatomical deadspace and functional residual capacity (FRC). We demonstrate that these parameters are robust and sensitive, and that they can distinguish between healthy individuals and those with mild-moderate COPD (stage 1–2), as well as distinguish between mild-moderate COPD (stage 1–2) and more severe (stage 3–4) COPD. In particular, σCL, a measure of unevenness in lung inflation/deflation, could represent a more sensitive non-invasive marker of early or mild COPD. In addition, by providing a multi-dimensional assessment of lung physiology, this technique may also give insight into the underlying pathophysiological phenotype for individual patients. These preliminary results warrant further investigation in larger clinical research studies, including interventional trials.

## Introduction

Chronic obstructive pulmonary disease (COPD) affects nearly 400 million people worldwide (Adeloye, et al., 2022) and is associated with high morbidity and mortality. It is characterised by airflow limitation, and is typically diagnosed by demonstrating obstructive spirometry in symptomatic patients, e.g. post-bronchodilation FEV_1_/FVC <0.70 (FEV_1_: forced expiratory volume in 1 s, FVC: forced vital capacity). Spirometry is also the main physiological measure used to assess severity and monitor progression in patients with COPD. However, while spirometry has been shown to be predictive of mortality and patient outcomes at a population level (Anthonisen, et al., 1986) there is often significant discord between FEV_1_ and symptoms, exacerbation frequency and disease progression at an individual level, perhaps reflecting the known heterogeneity in COPD pathophysiology (West, 2011). Furthermore, because FEV_1_ is substantially influenced by large airway function, significant disease will have accumulated in the smaller (<2 mm diameter) airways before a diagnosis of COPD can be made (McDonough, et al., 2011) (Johns, et al., 2014).

An alternative approach to physiological measurement in COPD is to focus on gas exchange inhomogeneity. The Multiple Gas Elimination Technique (MIGET), for example, has been used to demonstrate significant inhomogeneity in both ventilation and perfusion in the lungs of patients with COPD (Wagner, et al., 1977), and to define distinct “phenotypes” of ventilation-perfusion mismatch. Interestingly, this method has also revealed significant gas exchange inhomogeneity in patients with GOLD (Global initiative for chronic Obstructive Lung Disease) Stage 1, i.e., patients with mild COPD with a FEV_1_ > 80% predicted, which was disproportionately greater than the measured airflow limitation in those patients (Rodríguez-Roisin, et al., 2009). This is in keeping with COPD as a disease that initially involves the small airways, parenchyma and vessels, prior to spirometric disturbance (Rodríguez-Roisin, et al., 2009). Abnormalities of lung inhomogeneity have also been measured in patients with COPD using the multiple breath washout method (Verbanck, et al., 1998).

A novel method for the assessment of lung inhomogeneity, based on highly-accurate measurements of gas-exchange at the mouth has previously been published by our group (Mountain, et al., 2018). This non-invasive approach utilises a mechanistic model of the lung, the outputs of which are fit to very precise measurements of respiratory gas exchange made by a bespoke molecular flow sensor (Ciaffoni, et al., 2016). It provides multiple novel parameters that serve as indices of inhomogeneity in the lung. In particular, the method quantifies the standard deviations (SD) of the distributions of three physiological properties - alveolar compliance (inflation/deflation), anatomical deadspace and vascular conductance—that vary across the lung parenchyma. The model of the lung is based upon a “normal” lung, constraining the distributions of compliance and conductance to be unimodal. This method is simple to perform for operator and participant, and in a recent study provided repeatable indices of inhomogeneity that could reliably discriminate between six young healthy individuals (20–30 years), six older healthy individuals (70–80 years) and six patients with mild-to-moderate COPD (Mountain, et al., 2018).

To explore further the applicability of this new inhomogeneity test in patients with COPD, we used the approach in a cohort of patients with COPD of varying severity (according to GOLD stage) and age-matched healthy controls. We tested the tolerability of the measurements in patients with more severe COPD, examined the performance of our lung model in more obstructed lungs, and explored the relationship between the novel inhomogeneity parameters and traditional spirometric measures of disease severity.

## Methods

### Experimental methods

Thirty-six participants (10 controls, 26 patients with COPD) each underwent standard forced spirometry and lung inhomogeneity testing. The lung inhomogeneity test is a nitrogen multiple breath washout (MBW) test during which the volunteer breathes through a mouthpiece with their nose occluded for 15 min. For the first 10 min they breathe air, and for the final 5 min they breathe 100% oxygen. The mouthpiece is connected to the novel molecular flow sensor (MFS), which records the flux of respired gases (O_2_, N_2_, CO_2,_ and water vapour) every 10 ms. Peripheral oxygen saturation (SpO_2_) is recorded throughout using a pulse-oximeter (Masimo, Radical 7). Where two washout procedures were performed on the same individual (to assess intra-subject repeatability), these were separated by a 15-min recovery period to allow re-equilibration of the lung with room air. COPD patients were studied at a time of stability (i.e. not during an exacerbation). Informed written consent was provided by each volunteer and the study was approved by the South Central Oxford A Research Ethics Committee (17/SC/0172).

### Lognormal Lung Model

**FIGURE 1:**
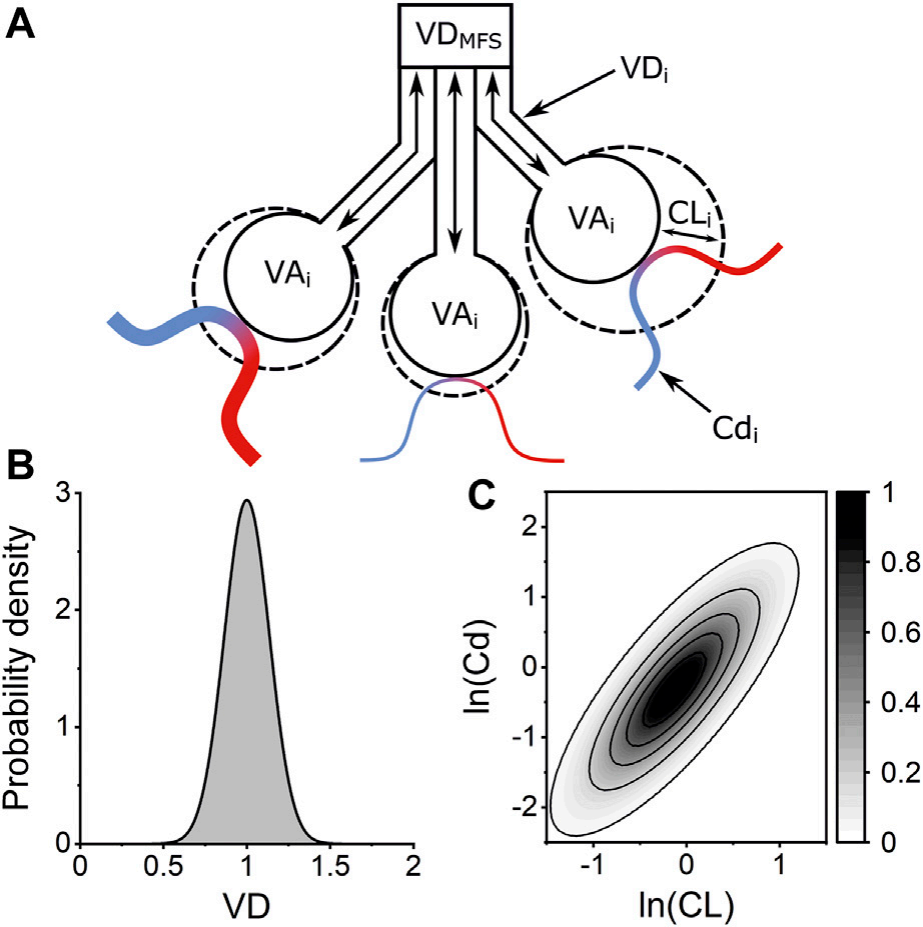
Lognormal Lung Model of pulmonary inhomogeneity. **(A)** Schematic of three lung units within the Lognormal Lung model. Each unit has the same alveolar volume at FRC (VA_i_) but the units vary in their fractional share of total anatomical deadspace volume (VD_i_), lung compliance (CL_i_), and vascular conductance (Cd_i_). Venous blood (blue) is oxygenated by the alveolar gas and departs as arterial blood (red). The anatomical deadspace introduced into the airway by the molecular flow sensor (VD_MFS_) is accounted for within the model. **(B)** Example of normal distribution used to describe the distribution of VD, the standardised deadspace to alveolar volume ratio, across the 125 lung units within the model. The width of this distribution is defined by the standard deviation, σVD, with an integrated area of 1. **(C)** Contour plot illustrating the bivariate lognormal distribution which describes the variation lung compliance (CL) and vascular conductance (Cd) across the lung units within the Lognormal Lung model. Contour intervals are values for the probability density function.

A computational model of an inhomogeneous lung, named the “Lognormal Lung Model,” was fit to the measured gas-exchange data. The model considers a total alveolar volume made up of 125 lung units. Each unit has equal alveolar volume at the lung’s functional residual capacity (FRC). The units vary in terms of their fractional share of total lung compliance, fractional share of total pulmonary vascular conductance, and fractional share of total deadspace. Alveolar compliance and vascular conductance are each assumed to be log-normally distributed across the 125 units with a coefficient of correlation set at 0.8. The fractional standardised deadspace is assumed to be normally distributed. A simplified schematic of the model is shown in Figure 1 along with illustrations of the distributions used to describe inhomogeneity within the lung. The model is fit to the measured N_2_ washout data by minimising the sum of squared differences between the model’s expired molecular gas flux values and those recorded experimentally. Three core indices of inhomogeneity are recovered: σCL, σCd, and σVD represent the standard deviations of the log of the standardised lung compliance, log of the standardised vascular conductance, and of the standardised deadspace all relative to lung volume, respectively. It also provides estimates of total anatomical deadspace (VDS) and alveolar volume (VA), and the FRC is calculated as the sum of the estimated VDS and VA.

The Lognormal Lung Model also makes an estimate of SpO_2_ for each individual washout. In cases where the modelled SpO_2_ was higher than the measured SpO_2_, this was attributed to the presence of pure shunt or lung units with very low ventilation:perfusion ratios; these features will not contribute significantly to the expired gas composition. However, if the modelled SpO_2_ was >3.5% lower than the measured SpO_2_, the associated recovered lung parameters were excluded from further analysis, reflecting the significant disparity between the modelled physiology and the individual’s true physiology.

To assess the quality of the Lognormal Lung Model’s fit to the measured data from each participant, the mean square error, MSE, of the differences between the measured and simulated molecular gas fluxes is evaluated. Further details of the Lognormal Lung Model are described elsewhere (Mountain, et al., 2018).

### Statistical analysis

The COPD patients were divided by GOLD Stage; 1: FEV_1_ > 80% predicted; 2: FEV_1_ 50%–79% predicted; 3: FEV_1_ 30%–49% predicted; and 4: FEV_1_ < 30% predicted. Due to the small number of available patients in Stage 1 and in Stage 4 (see Table 1), to evaluate whether the Lognormal Lung parameters could distinguish patients with more severe disease (including more obstructed lungs) from those with less severe disease using a statistical linear fixed-effects model, we grouped together patients in GOLD Stages 1 and 2, and patients in Stages 3 and 4. As such, we used a linear fixed-effects model in which “severity group” was a categorical fixed effect with 0: healthy control group, 1: GOLD Stage 1 and 2 patients; and 2: Gold Stage 3 and 4 patients. To control for possible effects of height, age, BMI, and sex on determined parameters, these participant characteristics were used as covariates in the model. For each determined parameter, *X*, the model was written as follows:

X=Intercept+αln(Height)+βln(Age)+γln(BMI)+δisFemale+Categorical[Severity Group]

(and in the case of volume parameters, e.g., FRC and deadspace volume, ln(*X*) was used).

**TABLE 1:** Anthropometric data for COPD patients and healthy controls. Data are shown as group mean ± standard deviation where applicable.

	Healthy controls (*n* = 10)	GOLD 1 (*n* = 4)	GOLD 2 (*n* = 10)	GOLD 3 (*n* = 10)	GOLD 4 (*n* = 2)	COPD all (*n* = 26)
Female# (%)	3 (30%)	2 (50%)	3 (30%)	2 (20%)	0 (0%)	7 (27%)
Age/years	69.5 ± 4.1	66.8 ± 12.3	68.4 ± 7.9	70.4 ± 6.3	57.5 ± 10.6	67.9 ± 8.9
Height/m	1.73 ± 0.09	1.65 ± 0.10	1.73 ± 0.12	1.68 ± 0.11	1.72 ± 0.09	1.70 ± 0.11
Weight/kg	71.4 ± 16.3	87.3 ± 28.5	85.0 ± 22.8	76.5 ± 22.3	92.7 ± 29.4	83.7 ± 23.9
BMI/kg m^−2^	23.1 ± 3.6	31.5 ± 7.7	28.2 ± 54	26.6 ± 5.7	31.1 ± 6.7	28.6 ± 6.1
FEV1/% pred	113 ± 10	93 ± 7	63 ± 7	39 ± 5	23 ± 1	55 ± 22
FVC/% pred	121 ± 11	114 ± 10	91 ± 20	83 ± 15	67 ± 3	89 ± 20
FEV1/FVC	0.74 ± 0.07	0.68 ± 0.09	0.53 ± 0.05	0.37 ± 0.07	0.27 ± 0.00	0.47 ± 0.14

Non-significant factors relating to participant characteristics were removed sequentially, least-significant first, until only the significant terms plus “Severity Group” remained.

Correlations were assessed using the Spearman rank-order method. Intra-participant parameter repeatability was assessed by calculating the standard deviation, σ, of the determined parameter values for each individual’s repeated tests. The average of these standard deviations was calculated across the group of subjects with COPD.

## Results

Anthropometric data and basic spirometry for the participants are shown in Table 1. The N_2_ washout experimental procedure was generally tolerated well by the patients with COPD. In order to assess intra-subject repeatability for patients with COPD, all patients were asked to perform two washout tests, but one patient chose to perform only a single inhomogeneity test. In two washout tests, the recorded N_2_ profiles indicated the presence of a leak from the participants’ nose or mouth, and in these cases the Lognormal Lung model failed to successfully fit to the measured gas-exchange data and to converge on an optimised set of parameters. In all other cases, the minimisation was successful, returning a set of estimates for the lung parameters. The computational time required to perform a successful minimisation was typically close to 1 hour. The parameters determined from a further seven datasets were excluded on the grounds that the modelled SpO_2_ during air breathing was more than 3.5% below the value measured by the pulse-oximeter during the same period. As such, there were two COPD patients for whom parameters from both experimental procedures were excluded and 24 for whom inhomogeneity parameters were obtained. For six of those participants a single parameter set was obtained, and for 18 participants two acceptable sets of parameters (from two washout tests) were available (in which case averaged values are presented).

Intra-subject parameter repeatability was assessed for the subjects with COPD for whom two accepted Lognormal Lung parameter sets were determined. The intra-subject repeatability of the airway parameters was good: the mean standard deviations of the determined volumes were 8.5% and 3.3% of mean parameter values for deadspace and alveolar volume, respectively; for σVD the mean standard deviation was 4.9% of the mean determined parameter, and for σCL this value was 5.2%. The variability was greater in the circulatory parameter, σCd, at 22.5% of the mean parameter value.

Linear fixed-effects modelling showed that the determined Lognormal Lung parameters for FRC and anatomical deadspace (VDS) were each significantly affected by BMI (*p* < 0.05 and *p* < 0.01, respectively), such that an increase in BMI was associated with a decrease in these parameters. Anatomical deadspace was also significantly increased by height (*p* < 0.05), whilst FRC was significantly lower for the female volunteers (*p* < 0.005). σVD and σCL were both significantly reduced by height (*p* < 0.05 in each case) and σCL also by age (*p* < 0.05). The mean square error, MSE, was significantly increased by height (*p* < 0.0005).

**FIGURE 2:**
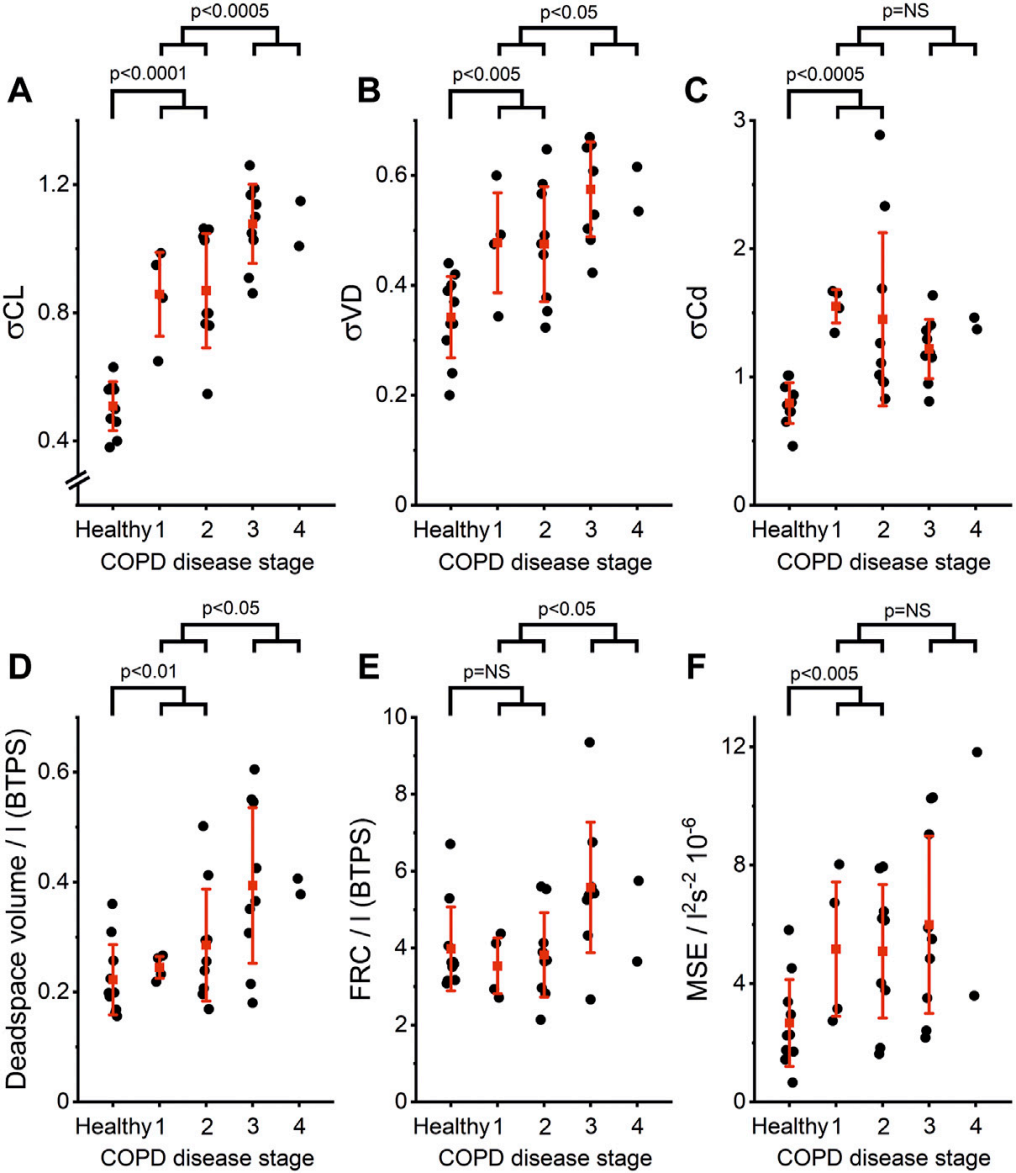
Lognormal Lung model parameters for healthy age-matched controls and COPD patients of increasing GOLD Stage. **(A)** Standard deviation for the distribution of lung compliance, σCL, **(B)** Standard deviation for the distribution of anatomical deadspace, σVD, **(C)** Standard deviation for the distribution of vascular conductance, σCd, **(D)** Anatomical deadspace volume **(E)** Functional residual capacity, FRC, and **(F)** Mean squared error, MSE. Gold Stages are defined as 1: FEV_1_ > 80% predicted; 2: 50% predicted < FEV_1_ < 80% predicted; 3: 30% predicted < FEV_1_ < 50% predicted; and, 4: FEV_1_ < 30% predicted. Data are shown for individuals in black, and stage-by-stage averages and standard deviations are shown in red. Group averages are not shown for stage 4 due to low group population. Significant differences were identified between healthy controls and patients with less severe obstruction (stages 1 and 2) for σCL, σCd, σVD, deadspace volume, and MSE. Significant differences between COPD patients with less severe obstruction (stages 1 and 2) and those with more severe obstruction (stages 3 and 4) were identified for σCL, σVD, deadspace volume, and FRC.

Figure 2 shows the determined Lognormal Lung parameters for patients in each COPD GOLD stage and for the healthy control group. Analysis with the linear fixed-effects model showed that σCL, σVD, and anatomical deadspace volume all significantly increased with increasing COPD severity: specifically, as shown in Figure 2, there were significant increases in group 1 (Stage 1–2) versus the control group (*p* < 0.0001 for σCL, *p* < 0.005 for σVD, and *p* < 0.01 for anatomical deadspace) and significant increases in group 2 (Stage 3–4) versus group 1 (*p* < 0.0005 for σCL, *p* < 0.05 for σVD and anatomical deadspace). For FRC, there was a significant increase in COPD patients with more severe disease (group 2) compared with those with milder disease (group 1) (*p* < 0.05) but no significant difference between the control group and group 1. For σCd and MSE there were significant increases in COPD group 1 versus the control group (*p* < 0.0005 and *p* < 0.005, respectively) but no significant difference between group 1 and group 2.

To gain some insight into whether the differences in parameters detected through linear regression were simply population or group differences, or whether it is possible to determine individually for patients with COPD if they were outside the normal range, we defined the upper limit of normal for each parameter as parameter mean + 1.98σ for the control group. We found that 22 of 24 COPD patients had σCL parameters greater than the upper limit of normal. This makes σCL a very sensitive marker for the presence of chronic airways disease that is meaningful at the level of an individual, and indeed it could distinguish GOLD stage 1 COPD patients from healthy controls in a direct pairwise comparison (*p* < 0.05, Welch *t*-test).

Finally, direct correlations between spirometric measurements (FEV_1_ %predicted and FEV_1_/FVC) and the Lognormal Lung inhomogeneity parameters within the COPD group were assessed by Spearman correlations (r). The strongest correlations were identified for σCL, for which there was a strong significant negative correlation with FEV_1_/FVC (r = −0.66, *p* < 0.0005), and a weaker, but nonetheless significant correlation with FEV_1_ %predicted (r = −0.61, *p* < 0.005). Significant negative correlations were also found for σVD with FEV_1_/FVC (r = −0.58, *p* < 0.001) and with FEV_1_ %predicted (r = −0.44, *p* < 0.05), but not for σCd.

## Discussion

In this study, a new non-invasive technique was used to assess lung inhomogeneity in a group of COPD patients with disease severity ranging from mild to very severe. Good patient tolerance of the N_2_ washout procedure demonstrated the clinical feasibility of this technique. The intra-individual repeatability of most of the determined parameters was very good. The exception was the repeatability of the recovered values for σCd, which were determined less precisely than the airway parameters, reflecting the inherent challenge of estimating vascular parameters from respiratory gas analysis.

All three inhomogeneity parameters (σCL, σVD, and σCd) were found to be significantly elevated in patients with COPD, compared to the healthy control group. This is consistent with previous literature where lung inhomogeneity is assessed by MIGET (Wagner, et al., 1977) and the multiple breath washout technique (Verbanck, et al., 1998). The advantage of our technique is that it is simple to perform and non-invasive, unlike MIGET, and that the parameters obtained relate to intrinsic physiological properties of the lung, unlike MBW-derived indices which rely on parameterisation of the nitrogen or tracer gas profiles at specific timepoints. As such, our technique has the potential not only to identify lung inhomogeneity, but also to provide important biological insights into the nature of the underlying lung abnormality.

Inherent within the Lognormal Lung model is the assumption of unimodal distributions of compliance and conductance across the lung units. Whilst this assumption can be confidently made in healthy individuals (Beck, et al., 2012), bi- or even trimodal distributions have been identified in patients with advanced COPD (Wagner, et al., 1977). This modelling assumption is likely to be the explanation for the model’s increased mean squared error for the COPD group, and may also underlie the difference observed for some patients between modelled and measured SpO_2_. Although this led to the exclusion of seven of the 49 determined parameter sets from our analysis, acceptable lung parameters were determined for 82% of lung inhomogeneity tests undertaken within the COPD patient group, and measurements were possible in 24 of the 26 patients (92%).

Importantly, we showed that the airway inhomogeneity model parameters, σCL and σVD, and the volume parameters for deadspace (VDS) and FRC were significantly higher in COPD patients with more severe lung obstruction compared to those with milder disease. We found that the airway parameter σCL, which describes the inhomogeneity of alveolar compliance (alveolar inflation/deflation), correlated with established spirometric measures of COPD disease severity (FEV_1_ %predicted and FEV_1_/FVC). However, these correlations are not perfect. There is significant overlap in σCL values between different COPD stages, and only 37% of the variance in σCL can be explained by FEV_1_%pred, and 44% by FEV_1_/FVC. This suggests that σCL is not merely a proxy for FEV_1,_ but may instead provide other valuable information about airways pathophysiology. One possibility is that σCL is a more sensitive marker than FEV_1_ or FEV_1_/FVC for disease burden in the small airways. This hypothesis is supported by the observation that σCL is significantly elevated in the stage 1 patient group, despite a normal FEV_1_ and an FEV_1_/FVC ratio that was only 3% below the normal threshold (0.7). In keeping with this, in a recent study of asthmatic patients, we demonstrated differential effects of bronchodilators on FEV_1_ and σCL, and showed that σCL was a better predictor of disease burden and control than FEV_1_, suggesting that it may be a surrogate marker of disease activity in the small airways (Smith, et al., 2020).

**FIGURE 3:**
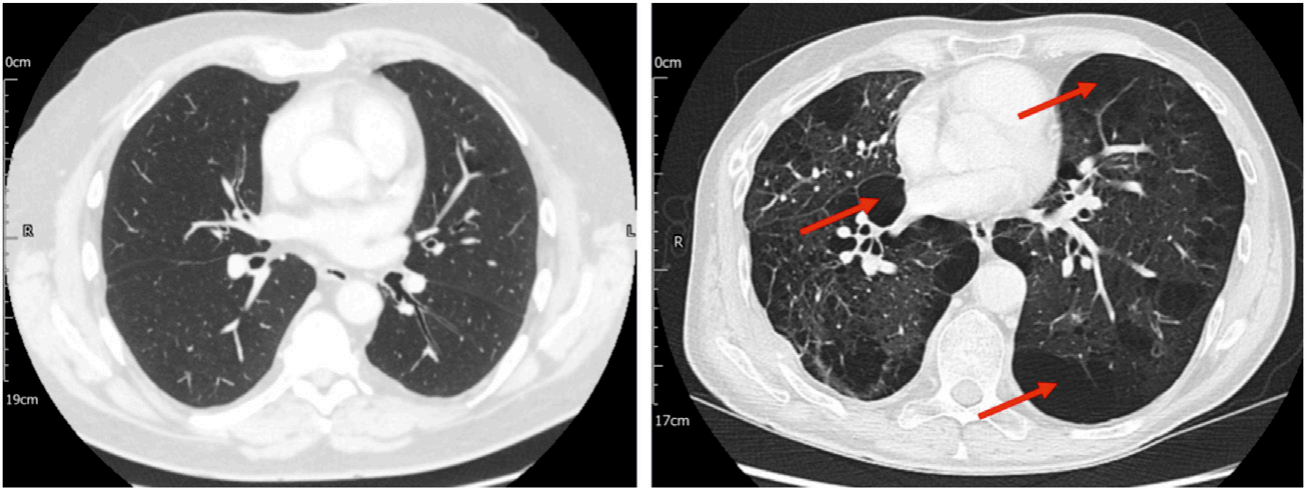
Computed tomography (CT) imaging for two patients with stage 2 COPD, one of whom had no CT evidence of emphysema (left panel) and the other of whom had significant paraseptal and centrilobular emphysema (right panel), with bullae (examples indicated by arrows). Both patients had significantly elevated σCL (0.76 and 1.06, respectively). In the patient without CT evidence of emphysema, the estimated total anatomical deadspace volume was normal (0.169 L). In the patient with CT evidence of emphysema, the estimated deadspace volume was substantially elevated (0.502 L). This highlights the possibility that lung inhomogeneity testing might not only represent a means of identifying COPD, but also of providing information about the underlying disease phenotype.

The assessment of anatomical deadspace is a physiological measure which is not currently measurable with routine lung function testing. The finding of increased anatomical deadspace seen in COPD patients is of pathophysiological and clinical interest. Notably, this index is not well correlated with spirometric indices of airflow obstruction and indeed there is a broad range of values within the middle stages of COPD with significant overlap between stages. One hypothesis is that our observations may reflect progressive centrilobular emphysema which acts to dilate and/or destroy the respiratory bronchioles (Hogg, 2004). Although too few patients had contemporaneous CT imaging in our current cohort to test this possibility formally, there were indications that this is worth exploring in larger datasets. Figure 3, for example, provides CT images for two patients with COPD. Both patients had a substantially elevated σCL, but the estimated anatomical dead space was elevated only in the patient with extensive emphysema.

Finally, our lung inhomogeneity test also returns an estimate of FRC, which was significantly higher in COPD patients with more severe obstruction (stages 3 and 4), compared with mild-moderate disease. This result is in keeping with the known tendency for lung hyperinflation in COPD, which may be attributed to reduced elastic recoil and expiratory flow limitation (O'Donnell & Laveneziana, 2006).

In summary, we present here the use of a new technique for assessing inhomogeneity in patients with COPD. We demonstrate that our novel physiological indices are robust and sensitive, suggesting that they may have clinical utility in the assessment and management of COPD. We also show that they can distinguish between patients with mild versus more severe disease, and importantly that they provide a multi-dimensional assessment of lung physiology including information about the underlying pathophysiology and phenotype of a patient. In particular, σCL could represent a useful non-invasive marker of early or mild small airways pathology, which could facilitate early intervention in these patients (Woodruff, et al., 2016). Larger clinical research studies, including interventional studies, will be required to evaluate its full potential as a method for comprehensive physiological phenotyping.

## Data Availability

The original contributions presented in the study are included in the article/supplementary materials, further inquiries can be directed to the corresponding author.
